# The Role of Hydrogen Bond Donors and Intramolecular Hydrogen Bonding in Modulating Blood–Brain Barrier Permeability: Implications for CNS Drug Design

**DOI:** 10.3390/molecules31132366

**Published:** 2026-07-05

**Authors:** Manuel Novás, Nuria Blanco López, Lourdes Santana, Eugenio Uriarte, Maria João Matos

**Affiliations:** 1 Departamento de Química Orgánica, Facultad de Farmacia, Universidade de Santiago de Compostela, 15782 Santiago de Compostela, Spain; manuel.novas.gonzalez@rai.usc.es (M.N.); nuria.blanco.lopez@rai.usc.es (N.B.L.); lourdes.santana@usc.es (L.S.); eugenio.uriarte@usc.es (E.U.); 2 Biofarma Research Group, Centro de Investigación en Medicina Molecular y Enfermedades Crónicas (CiMUS), Universidade de Santiago de Compostela, 15782 Santiago de Compostela, Spain

**Keywords:** hydrogen bond donors, intramolecular hydrogen bonding, blood–brain barrier, permeability, central nervous system, drug design

## Abstract

The blood–brain barrier (BBB) represents a major challenge in central nervous system (CNS) drug development due to its selective permeability. Multiple molecular properties such as lipophilicity, molecular size, and hydrogen-bonding potential critically influence a compound’s ability to cross the BBB. In particular, the presence of hydrogen bond donors (HBDs) and the ability to form intramolecular hydrogen bonds (IMHBs) play a crucial role in modulating brain penetration. This review discusses the mechanistic impact of HBDs and IMHBs on BBB permeability, highlighting key physicochemical parameters and case studies that demonstrate the utility of IMHBs in CNS drug design. Strategies to mask polar functionalities through IMHB formation are emphasized as promising tools to optimize brain delivery without compromising pharmacological activities. The literature reviewed in this work was collected through comprehensive searches of scientific databases, including SciFinder, PubMed, Web of Science, and Scopus, complemented by manual search of reference lists from relevant publications.

## 1. Introduction

The blood–brain barrier (BBB) is a highly selective, semipermeable membrane that protects the central nervous system (CNS) from potentially harmful substances while allowing essential nutrients to pass through [1]. This physiological barrier presents a robust obstacle for the delivery of therapeutic agents to the brain. While many small-molecule drugs are efficacious in peripheral targets, their effectiveness in CNS applications is often limited by poor BBB penetration [2].

Physicochemical properties, such as molecular weight, lipophilicity, polar surface area (PSA), and hydrogen-bonding capacity, are critical in determining BBB permeability. Among these, hydrogen bond donors (HBDs) [3] and the potential for intramolecular hydrogen bonding (IMHB) play key roles in modulating molecular polarity and membrane diffusion [4,5]. IMHB formation can reduce exposed polarity and improve passive permeability, but BBB penetration depends on multiple additional factors, including ionization state, p*K*a, log*D*, conformational equilibrium, solubility, metabolic stability, and transporter-mediated efflux. IMHBs are often exploited in drug design to mask polar groups, improve membrane permeability, and pre-organize ligands for receptor binding. Therefore, HBDs and IMHBs play an important role in the ability of molecules to cross the BBB, which is a key consideration in CNS drug design [6].

**Figure 1:**
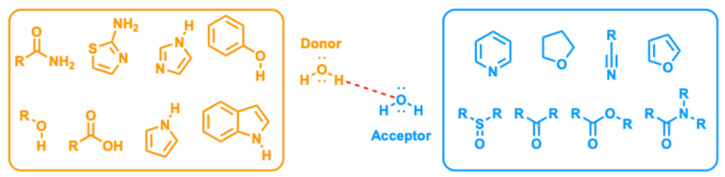
Examples of hydrogen bond donors (HBDs) and acceptors (HBAs) especially relevant in drug discovery.

Due to its physicochemical nature, the BBB strongly limits the passage of hydrophilic, highly hydrogen-bonding molecules, while favoring the passive diffusion of small lipophilic or amphiphilic compounds [7]. The number and nature of HBDs significantly influence a molecule’s ability to cross the BBB [8]. Molecules with a high number of HBDs tend to form strong interactions with water, increasing their polarity and reducing passive diffusion through the lipid-rich environment of the BBB [9]. As a general rule, BBB-permeable compounds typically possess ≤ 1–2 HBDs, and low polar surface area (PSA). However, compounds that form IMHBs can mask their polarity, effectively reducing the number of exposed HBDs or acceptors (HBAs) (Figure 1) [10]. This polar masking effect allows the molecule to maintain CNS activity despite having functional groups that would otherwise hinder BBB penetration. IMHBs can improve membrane permeability by enabling molecules to adopt a more lipophilic conformation in nonpolar environments such as the BBB [11].

Partitioning to different membranes requires for several molecules that a conformation can be adopted, which is also populated in the membrane interior. For the closed conformations, the polar groups become shielded from the apolar environment, typically through IMHB, facilitating improved membrane permeability and intestinal absorption [12,13]. A closed conformation in a polar environment is also important, as the rigidity of the scaffold can be beneficial to increase the permeability [14]. However, an increase in rigidity may be a handicap, as it may affect solubility and prevent interactions with the intracellular targets [15]. Therefore, an equilibrium should be achieved, and some conformational flexibility is necessary to reduce the energetic penalty, both enthalpic and entropic, associated with insertion into the membrane [16]. This chemical behavior is not easily designed, being more common in natural products. Therefore, these are usually more willing to a good passive permeability [17].

Based on the above-mentioned, in CNS drug design, reducing the number of free HBDs, and/or strategically designing molecules capable of forming IMHBs, is a key strategy to enhance passive diffusion across the BBB while maintaining pharmacological activity. Compounds with many HBDs usually have low passive permeability, and are not inherently substrates of uptake transporters, unless they are deliberately designed as transporter mimetics or prodrugs. Without a suitable transporter, such compounds typically exhibit limited membrane crossing and may require alternative strategies (e.g., prodrug approaches, HBD masking, or formulation-based delivery) [3].

This paper explores the contribution of HBDs and IMHBs in modulating the physicochemical properties and BBB permeability, focusing on their mechanistic implications and relevance to rational drug design. Both traditional *rule-of-five*-compliant compounds and selected *beyond-rule-of-five* scaffolds are considered when relevant to central CNS exposure and drug design. This review is primarily focused on small-molecule drugs, whereas biologics, peptides, and other macromolecular therapeutic modalities are not covered. While previous reviews have discussed CNS multiparameter optimization and general determinants of brain penetration, as well as the role of IMHBs in membrane permeability, a more integrated discussion specifically linking IMHB formation to BBB transport and CNS exposure remains less developed. Here, we aim to bridge this gap by critically examining how IMHB formation influences key properties such as polarity, conformational behavior, and passive permeability, while also highlighting its limitations within the broader multiparameter context that governs CNS drug accumulation.

For the preparation of this review, the relevant literature was identified through comprehensive searches in SciFinder, PubMed, Web of Science, and Scopus, and further complemented by manual examination of reference lists from selected publications. Searches were performed using combinations of keywords including “intramolecular hydrogen bond”, “IMHB”, “hydrogen bond donor”, “CNS drug design”, “membrane permeability”, “blood–brain barrier”, “CNS penetration”, and related terms. The review primarily focuses on publications from the last ten years to reflect recent advances in the field, although earlier seminal studies were included where necessary to provide historical perspective and mechanistic foundations. Articles were selected based on their relevance to the role of IMHBs in influencing physicochemical properties, permeability, pharmacokinetics, and medicinal chemistry applications.

## 2. Hydrogen Bond Donors and BBB Permeability

HBDs are atoms or groups capable of donating a hydrogen atom to form hydrogen bonds with HBAs, typically oxygen or nitrogen atoms. In aqueous environments, HBDs increase water solubility through strong interactions with water molecules. However, this increased polarity is detrimental to passive diffusion across lipid membranes such as the BBB.

As said before, empirical studies suggest that CNS-active drugs typically have ≤2 free HBDs. An excess of HBDs leads to increased interaction with the aqueous extracellular environment and impedes membrane permeability. The *rule of five* by Lipinski, although primarily for oral bioavailability, is echoed in CNS design by the *rule of three*, which further restricts parameters, including the number of HBDs [18]. Nuclear magnetic resonance (NMR), infrared and Raman, microwave, diffraction (X-ray and neutron diffraction) and calorimetry techniques serve as evidence for IMHB in several *beyond-rule-of-five* oral drugs [19].

The introduced HBDs are intended to, and do, participate in pharmacodynamic interactions with the target sites. Although HBDs can negatively impact permeability, once the compounds reach the target environment, these groups are available to form productive hydrogen-bond interactions with key amino acid residues in the binding site. Molecular docking and molecular dynamics simulations support that the HBDs contribute to binding stabilization and target recognition, for example by engaging catalytic or anchoring residues through hydrogen bonding. Thus, the HBDs play a functional pharmacodynamic role, even if they are partially masked during membrane transit, such as via IMHB formation [20].

## 3. Polar Surface Area (PSA) and Topological Polar Surface Area (tPSA)

PSA refers to the total surface area of a molecule occupied by polar atoms, typically oxygen and nitrogen, including their attached hydrogen atoms. It is a widely used descriptor in medicinal chemistry to estimate a compound’s ability to cross biological membranes, such as the intestinal epithelium or the BBB. PSA is directly influenced by the presence of hydrogen-bonding groups. CNS drugs generally exhibit a PSA of <90 Å^2^, as higher values correlate with poor BBB penetration. For optimal BBB penetration, some studies suggest a stricter threshold of <70–75 Å^2^. According to Lipinski’s seminal *rule of five*, compounds with more than five HBDs are more likely to exhibit poor absorption or permeation, and limiting the number of HBDs is one of the empirical criteria associated with improved oral bioavailability and, indirectly, with lower overall polarity and PSA in orally absorbed drugs [21].

For a compound to passively diffuse through the BBB, it must have limited polarity to move through the lipid-rich endothelial cell membranes. Drugs with PSA > 120 Å^2^ generally do not cross the BBB via passive diffusion. Molecules with high PSA tend to be more soluble in water, form strong hydrogen bonds with aqueous environments and have reduced permeability through lipophilic barriers. Therefore, a low PSA is favorable for CNS penetration [22].

HBDs (e.g., OH, NH_2_) and HBAs (e.g., carbonyl, ether, nitro) contribute to the PSA. Avoid implying that reducing HBDs alone will necessarily lower PSA to acceptable levels. Each HBD adds roughly 15–20 Å^2^ to the total PSA, depending on the molecular context. Therefore, reducing the number of HBDs in a molecule can substantially lower the PSA, enhancing its potential to passively cross the BBB [23].

While topological PSA (tPSA) is a 2D, structure-based estimation, the actual PSA (3D) can be lower if the molecule forms IMHBs. This masks polar functional groups, effectively reducing their solvent exposure and apparent polarity. This explains why some CNS-active drugs exceed the ideal tPSA threshold yet still penetrate the BBB due to favorable conformational effects and polar masking. This is why tPSA is commonly used as a filter in early-stage CNS drug discovery, even when big libraries of compounds are being screened. Many CNS-focused screening libraries apply, as said before, a cutoff of 90 Å^2^ to identify brain-penetrant candidates.

## 4. Log*P* and IMHBs in CNS Drug Discovery

The design of drugs for CNS disorders requires careful optimization of physicochemical properties to ensure effective brain penetration. Among these properties, the partition coefficient, expressed as log*P*, remains one of the most informative parameters. Log*P* reflects the balance between lipophilicity and hydrophilicity, and it is closely linked to a compound’s ability to cross biological membranes, including the BBB [24].

An additional layer of complexity arises from the role of IMHBs. When polar groups within a molecule form hydrogen bonds internally, their ability to interact with water or membrane surfaces decreases. This intramolecular masking of polarity reduces the apparent polarity of the compound, sometimes allowing molecules with seemingly unfavorable polar surface area or hydrogen bond counts to penetrate the BBB more effectively.

In this context, log*P* becomes a valuable indicator of how IMHBs influence membrane permeability. Molecules that form stable IMHBs may exhibit log*P* values that suggest higher lipophilicity than would be expected based on their structural features alone. This “hidden polarity” can be advantageous in CNS drug design, where a delicate balance is needed: compounds must be lipophilic enough to cross the BBB, but not so lipophilic that solubility or selectivity is compromised [5].

Therefore, considering both log*P* and the potential for IMHB formation offers medicinal chemists a more nuanced framework for predicting CNS permeability. By integrating these factors, researchers can better design candidate molecules that achieve the desired pharmacokinetic profile while maintaining favorable pharmacodynamics for the treatment of CNS diseases. Nevertheless, the influence of IMHB formation on BBB penetration should be considered within the broader context of molecular and biological determinants of drug delivery. The impact of IMHB formation depends on a complex interplay of factors, including ionization state, lipophilicity, conformational dynamics, metabolic stability, and transporter interactions. Furthermore, the reduction in exposed polarity achieved through IMHB formation may be accompanied by decreased aqueous solubility, potentially compromising overall drug accumulation in the brain. IMHB-induced conformational constraints may also negatively affect target recognition, binding affinity, or the ability of a molecule to adopt bioactive conformations. Moreover, for compounds that rely on carrier-mediated uptake mechanisms, transporter activity may be a more important determinant of permeability and tissue distribution than IMHB formation itself. Consequently, IMHBs should be viewed as one component of a broader multiparametric optimization strategy rather than as a universal solution for improving CNS exposure or pharmacokinetic properties.

## 5. Intramolecular Hydrogen Bonding as a Masking Strategy for CNS Drug Design

IMHB refers to hydrogen bonds formed within the same molecule. These bonds can effectively mask polar groups, reduce the overall solvent-accessible surface area, and facilitate a more lipophilic conformation in nonpolar environments like the BBB [25].

The mechanistic benefits of IMHBs are the reduction in effective polarity (IMHBs shield HBDs from solvent exposure), conformational restriction (favors low-energy conformers that diffuse more easily through membranes) and dynamic polarity switching (molecules may adopt polar conformations in aqueous environments and nonpolar ones in membranes, enhancing transport) [26].

To optimize brain penetration without sacrificing activity, medicinal chemists employ strategies such as reduction of free HBDs (e.g., masking hydroxyls or amines), incorporation of IMHB-capable motifs (e.g., *ortho*-substituted phenols, amides adjacent to HBAs) and prodrug approaches, to temporarily mask polar groups. Computational tools, such as 3D PSA calculations, molecular dynamics, and conformational sampling, can predict IMHB formation and its effect on permeability.

**Figure 2:**
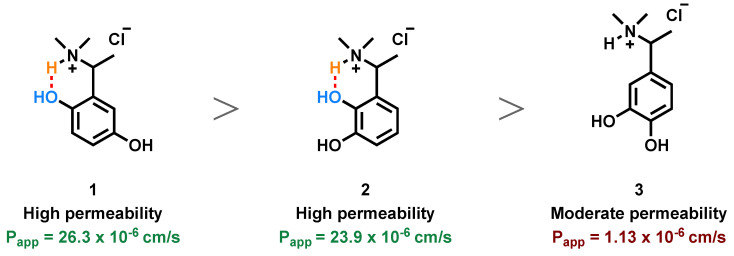
Example of how IMHBs improve BBB penetration of two described Aβ42 fibrillation inhibitors IMHBs. Experimental evidence reported in Dighe et al. [27].

As previously described by Dighe et al., IMHBs can shield polar hydroxy and tertiary amine groups (e.g., compounds of compounds **1** and **2**, Figure 2). These molecules show higher permeability than compound **3** (Figure 2), which does not form IMHBs [27]. While the experimental studies indicate improved membrane permeability, the exact protonation states during permeation have not been directly measured. These compounds exhibit potent Aβ42 fibrillation inhibition and favorable membrane permeability profiles and have been proposed as multifunctional leads for Alzheimer’s disease therapy.

Enhanced brain penetration and pharmacological efficacy have been reported for NK1 receptor antagonists [28]. It has been suggested that IMHB formation may contribute to these properties by reducing the apparent polarity of the molecules, potentially increasing their effective lipophilicity. Researchers at Takeda demonstrated that establishing such IMHBs in luteinizing hormone-releasing hormone receptor antagonists led to improved oral bioavailability and favorable pharmacokinetic properties [29]. Investigations involving cyclic peptides further support the importance of IMHBs in enabling passive membrane permeability. Interestingly, the geometries of IMHBs in small-ring systems often deviate from those commonly seen in flexible, unconstrained molecules.

A recent investigation on psilocin provided experimental evidence of IMHB formation, thereby confirming the long-standing hypothesis that its oral activity and psychoactive potency are closely linked to unique IMHB interactions. In particular, the ability of psilocin to adopt a pseudo-ring conformation through an O–H···N intramolecular bond carries important implications for its pharmacological and pharmacokinetic properties [30]. First, stabilization through the intramolecular O–H···N hydrogen bond reduces the likelihood of N-protonation and O-deprotonation, as the corresponding ionized forms do not receive equivalent energetic compensation. This effect offers a plausible rationale for the reduced basicity reported for psilocin. As a result, a larger fraction of the compound remains uncharged, facilitating transfer from aqueous physiological environments into lipophilic membranes such as the BBB. Second, by internally shielding its most polar moieties, psilocin effectively increases its solubility in nonpolar media, further enhancing membrane partitioning. Third, the pseudo-ring conformation created by this IMHB may also account for the relatively slow metabolic degradation of psilocin by monoamine oxidase (MAO). In contrast, structurally related compounds that are unable to form IMHBs are generally more polar, exhibit higher susceptibility to MAO-mediated breakdown, and show limited ability to cross the BBB. This explains why exogenous 5-hydroxytryptamines, although potent ligands at 5-HT receptors in vitro, fail to act as psychoactive agents in vivo [31]. On the other hand, non-hydroxylated tryptamines such as *N*,*N*-dimethyltryptamine (DMT) and 5-methoxy-DMT possess sufficient lipophilicity to cross the BBB but are rapidly metabolized by MAO, rendering them orally inactive without MAO inhibition. Collectively, these findings provide deeper insight into the distinctive pharmacological profile of psilocin, a naturally occurring monomeric psychedelic that is currently under intense investigation as a promising therapeutic candidate for major depressive disorder [32].

**Figure 3:**
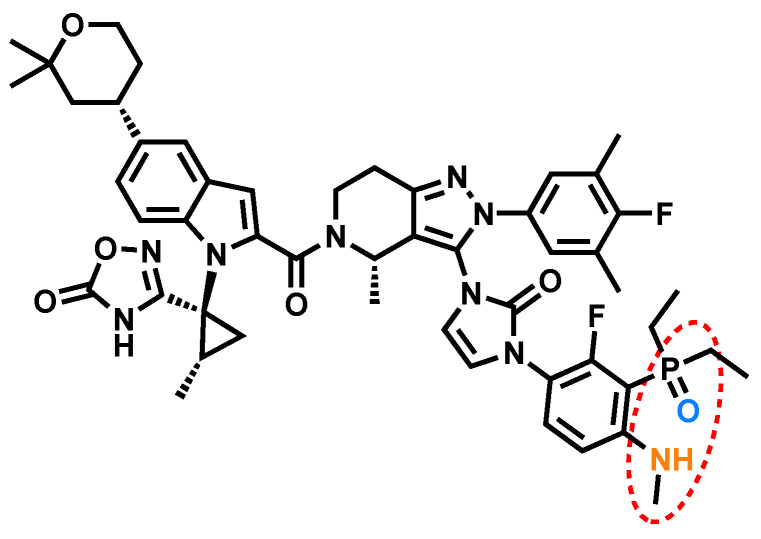
Structure of aleniglipron, highlining the IMHB site.

Aleniglipron (Figure 3) is an orally bioavailable small-molecule glucagon-like peptide-1 receptor (GLP-1R) agonist developed by Structure Therapeutics [33]. As of 2023, it has advanced into Phase II clinical evaluation. In June 2024, Structure Therapeutics announced positive topline results from a Phase IIa study in patients with obesity, where GSBR-1290 achieved clinically meaningful and statistically significant placebo-adjusted reductions in body weight. The compound was also reported to exhibit a generally favorable safety and tolerability profile. The presence of an IMHB site can be a key feature that influences its physical and biological properties of aleniglipron. IMHB are proposed to modulate lipophilicity by shielding polar groups from the aqueous environment, potentially facilitating diffusion across biological barriers such as the BBB [34]. Direct experimental validation of their effect on BBB permeability for aleniglipron has not yet been published. Therefore, our discussion focuses on the mechanistic rationale suggested by structure and predictions, rather than definitive experimental measurements.

Regarding the approved drugs, in Table 1 are represented some examples of drugs that despite containing polar functional groups, demonstrate brain penetration due to conformational masking of HBDs and HBAs via IMHB formation. Structural and biophysical studies support the presence of IMHBs in several cases.

Compounds such as lopinavir and ritonavir, known HIV protease inhibitors, demonstrate the importance of IMHBs in achieving CNS penetration despite having polar groups. Similarly, the design of CNS-penetrant kinase inhibitors often incorporates functional groups capable of forming IMHBs to balance potency and permeability [35].

Loperamide, an antidiarrheal, despite being a P-glycoprotein (P-gp) substrate and having multiple polar groups (e.g., amide, phenolic OH), is not CNS-active under normal circumstances because of efflux, but structurally, it is BBB-permeable. Generally, P-gp transports hydrophobic and weakly cationic substrates [36]. This molecule forms an IMHB between the oxygen of the tertiary amine and the hydrogen on the hydroxyl group on the opposite side of the molecule, which plays a role in its passive diffusion across cell membranes. IMHBs between the amide NH and adjacent HBAs have been proposed to reduce PSA and mask polarity, contributing to its inherent CNS permeability. This internal bond helps the molecule maintain a more lipophilic state by reducing the interaction of its polar groups with water, a process known as desolvation. This is why, in P-gp knockout models, loperamide induces CNS effects like opioid analgesia. Orally administered loperamide does cross the intestinal epithelium, but its systemic exposure is limited by first-pass metabolism (mainly CYP3A4 and CYP2C8) and efflux transporters, further reducing systemic bioavailability. Loperamide is highly lipophilic, allowing it to passively diffuse across the intestinal epithelial cells. Its permeability in Caco-2 cell models is high, indicating efficient uptake from the gut lumen into enterocytes [37]. Loperamide is also a substrate of intestinal P-gp (MDR1), as discussed before, which actively pumps some of the absorbed drug back into the lumen. This may limit the fraction that enters the portal circulation. Although loperamide crosses the gut epithelium, very little reaches the brain because it is also a high-affinity P-gp substrate at the BBB, which actively effluxes it back into the blood. This is why loperamide acts peripherally as an antidiarrheal with minimal central opioid effects [37].

Cyclosporin A, an immunosuppressant with a molecular weight ~1200 Da, despite its large size and many polar groups, can cross the BBB. It adopts a compact, folded conformation with multiple IMHBs, which mask polar functionalities and result in low effective PSA [38]. While IMHB formation can enhance membrane permeability by reducing the apparent polarity of a molecule, it should not be considered sufficient on its own to guarantee CNS exposure. Other factors, particularly active efflux mechanisms at the BBB, can significantly limit brain penetration, as particularly illustrated by compounds such as loperamide and cyclosporin A, which are efficiently transported by P-gp despite their ability to form IMHBs.

**Figure 4:**
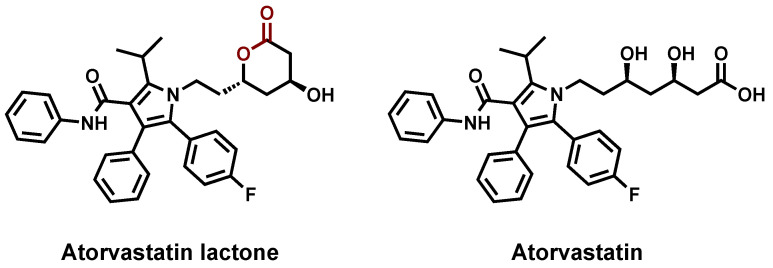
Structures of atorvastatin lactone prodrug and atorvastatin active drug.

Atorvastatin, in its lactone form (Figure 4), is a lipid-lowering agent. The acid form of atorvastatin has poor BBB permeability due to its high polarity. The lactone prodrug form neutralizes the anionic carboxylate group, reducing polarity and enabling improved membrane permeability. While IMHB is formed, there is currently no direct evidence that it *per se* contributes to CNS penetration. Thus, the enhanced permeability of lactone prodrugs is primarily attributable to carboxylate neutralization, though IMHB formation could potentially provide additional, reversible masking of polar groups [39].

Finally, sildenafil, a PDE5 inhibitor, contains amide and heteroatoms, yet has documented CNS effects [40,41]. Experimental X-ray and computational studies show conformers stabilized by IMHBs that reduce solvent exposure of HBDs and HBAs. X-ray analysis of sildenafil in its citrate salt form shows a predominantly planar conformation, stabilized by an intramolecular interaction between the pyrimidinone NH and the ethoxy moiety [42].

In summary, there are some known P-gp substrates that form IMHBs. It is the case for loperamide, that can form IMHDs that mask polarity and enhance membrane partitioning, yet is a classic P-gp substrate. Its exclusion from the brain is primarily due to active efflux by MDR1, despite favorable passive permeability. Cyclosporin A forms extensive IMHB networks and is highly lipophilic, but it is both a substrate and inhibitor of P-gp, demonstrating that IMHBs do not prevent transporter interaction. This drug demonstrates that BBB penetration is a balance between passive permeability and active efflux. It can cross the endothelial membrane, but MDR1 prevents its accumulation in the brain. This example is often cited to illustrate why good lipophilicity and IMHB formation alone are insufficient for CNS exposure if active efflux dominates [38].

Another good example is verapamil, which can adopt IMHB-stabilized conformations and is a well-established P-gp substrate, commonly used as a reference compound in efflux assays. P-gp recognition is not driven only by exposed polarity or hydrophilicity. Instead, substrates typically share sufficient lipophilicity to partition into the membrane, conformational flexibility, and appropriate HBAs patterns, which remain available even when HBDs are transiently masked by IMHBs. Thus, while IMHBs reduce effective polarity and favor passive diffusion, they do not eliminate molecular features required for P-gp binding. IMHB formation can enhance membrane permeation, but does not prevent compounds from being MDR1 (P-gp) substrates, as demonstrated by multiple clinically relevant examples and transporter assays [43].

**Figure 5:**
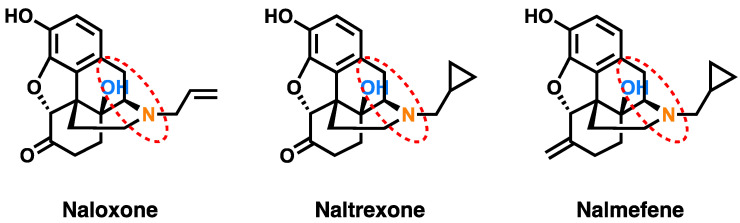
Structure of naloxone, naltrexone and nalmefene, highlining the IMHB site.

Three FDA-approved opioid antagonists, naloxone, naltrexone and nalmefene, have been recently studied to understand the impact of IMHBs on their activity and pharmacokinetics [44]. Naloxone, naltrexone, and nalmefene (Figure 5) possess a common morphinan framework enriched with polar groups that can engage in IMHBs. IMHB formation transiently masks polar functionalities, reducing the effective polar surface area and desolvation penalty, which facilitates transcellular passive diffusion across the lipid bilayer. Once inside the aqueous intracellular environment, these interactions can be disrupted, allowing the compounds to re-expose polar groups and engage their biological targets.

The exhaustive and complete theoretical and experimental work on these drugs and some derivatives completely clarified these thoughts. Differences in how polarity is expressed *versus* internally shielded by IMHBs are proposed to contribute to the fast CNS penetration of naloxone in contrast to the extended half-lives observed for naltrexone and nalmefene, underlining the relevance of IMHBs as structural determinants of both efficacy and duration of therapeutic action [44].

## 6. Limitations and Trade-Offs of IMHB in BBB Penetration

Although IMHBs can reduce exposed polarity and facilitate passive membrane permeation, their presence alone does not guarantee successful BBB penetration. As previously stated, brain exposure is governed by multiple physicochemical and biological factors, including ionization state, lipophilicity, molecular size, conformational dynamics, metabolic stability, and interactions with membrane transporters. In addition, the structural and functional integrity of the BBB may be impaired during neurodegenerative diseases. These processes are often associated with BBB dysfunction, which can alter its permeability and transport properties [45,46]. Consequently, the beneficial effects of IMHB formation on passive permeability may not necessarily translate into increased CNS exposure.

A major limitation is that compounds capable of forming IMHBs may still be subject to active efflux at the BBB. For example, molecules such as loperamide and cyclosporin A exhibit properties consistent with IMHB formation, yet their brain accumulation is significantly restricted by P-gp-mediated transport. These examples highlight that transporter-mediated efflux can override the favorable effects of IMHBs on passive diffusion.

Furthermore, the physicochemical changes associated with IMHB formation may involve trade-offs. Shielding polar functionalities often increases the apparent lipophilicity of a molecule, which can lead to reduced aqueous solubility and potentially compromise absorption, formulation, or systemic exposure. In addition, IMHBs can stabilize specific conformations that may not always be optimal for target recognition, potentially affecting biological activity or binding affinity. For compounds that rely on carrier-mediated uptake mechanisms, transporter interactions may play a more decisive role in tissue distribution than passive permeability enhancements resulting from IMHB formation.

Despite their well-documented benefits in reducing exposed polarity and enhancing passive membrane permeability, IMHBs are not universally advantageous and should not be regarded as a standalone strategy for optimizing BBB penetration. Therefore, IMHBs should be considered as one element within a multiparametric medicinal chemistry optimization strategy. Their contribution to BBB penetration and CNS exposure must be evaluated in conjunction with other molecular properties and biological processes that collectively determine drug disposition.

## 7. Biological Relevance of IMHB and BBB-Permeability Determination

There are several ways to determine the formation of IMHB which may lead to varying levels of biological relevance of the results, depending on the techniques used. NMR techniques are the most common tools and consist of analyzing ^1^H NMR chemical shifts in deuterated chloroform (CDCl_3_) and dimethyl sulfoxide (DMSO-d_6_) of OH and NH groups [47] or reduction of deuterium exchange rates [48], among others. NMR IMHB measurements are typically performed in CDCl_3_ or DMSO-d_6_ which are aprotic, non-competitive solvents. These may dramatically overestimate IMHB stability compared to what occurs in aqueous, competitive biological environments (cytoplasm, blood or interstitial fluid). Water molecules actively compete and disrupt IMHB. NMR in organic solvents removes this competition entirely, so an IMHB that appears robust in CDCl_3_ may be broken in aqueous media. Infrared spectroscopy (IR) determination can also be used but it requires non-hydrogen-bonding solvents, thus having the same disadvantage previously mentioned, making it poorly suited for drug discovery purposes [49].

X-ray and neutron diffraction may also be used, the former being less preferred as it has severe limitations in determining the position of hydrogen atoms [49]. When X-ray diffraction is employed, hydrogen positions are either not resolved or must be inferred computationally because of their low electron density. Since the hydrogen atom is the actual donor in a hydrogen bond, this is a fundamental limitation. Furthermore, the crystal lattice may force intermolecular contacts that do not exist in solution or in vivo. These packing forces can stabilize or distort conformations including hydrogen bond geometries that are not representative of the free molecule in a biological environment. Therefore, an IMHB observed in the crystal may be an artifact of crystal contacts, or a genuine IMHB may be broken by them [50]. Moreover, X-ray diffraction gives a snapshot of the electron density at one point in time. Biology is dynamic because proteins, peptides, and small molecules fluctuate between conformations constantly. This technique cannot capture this conformational variability, so the observed IMHB may represent only the lowest-energy crystal-state geometry, not the dominant solution conformer.

Evaluating BBB permeability in vitro is also a challenge. Parallel artificial membrane permeation assay (PAMPA) has been widely used and even adapted for BBB-permeation studies, implementing the use of polar brain lipid fractions to create a lipid bilayer that resembles that of the brain [51]. Nevertheless, this assay only accounts for passive diffusion. Drugs that are substrates of efflux transporters may have a higher predicted BBB permeability in a PAMPA while their in vivo bioavailability in the brain may be lower. Likewise, drugs that cross the BBB thanks to active transporters may have low permeation in a PAMPA while having a high brain bioavailability in vivo. Caco-2 or MDCK-MDR1 are cell-based permeability assays that allow us to determine whether drugs are substrates of P-gP or other efflux transporters. Nevertheless, these non-neuronal cell lines (human colorectal adenocarcinoma and canine cells, respectively) are not the best representation of the BBB, comprised mainly of neurons and glial cells. For that reason, combining data from a PAMPA-BBB assay with that of one of the aforementioned cell-based assays or using primary brain endothelial cells (BECs) has been shown to be a promising strategy to determine human BBB permeation. Cellular models can be either 2D or 3D and static or dynamic. 3D dynamic models are probably the closest to in vivo results, especially when used in organ-on-a chip models as they implement the shear stress component produced by blood in the brain vessels. Despite the advances in in vitro models, in vivo rat models still prevail as the most widely used in preclinical CNS research. However, they fail to account for the full complexity of human diseases, due to species-specific BBB differences in morphology and gene expression [52].

## 8. Conclusions

The number of HBDs and the potential to form IMHBs appear to be critical determinants of BBB permeability. Reducing the number of free HBDs and leveraging IMHBs offers effective strategies to design CNS-active drugs with adequate brain penetration. A deeper understanding of these molecular features, supported by computational and experimental validation, can significantly enhance the success in CNS drug discovery.

## Figures and Tables

**Table 1:** Examples of CNS-active drugs that display IMHB as a strategy to reduce effective polarity and enhance BBB permeability.

Drug	Polar Groups	IMHB Role	BBB Permeability
Lopinavir	Hydroxyl, amide, amine	OH ↔ amide C=O; conformational IMHB	Yes (partially P-gp effluxed)
Ritonavir	Hydroxyl, thiazole, amide, urea	OH/NH ↔ carbonyl acceptors	Yes (despite high polarity)
Loperamide	Amide, Hydroxyl	Amide NH ↔ nearby O	Yes (if not effluxed)
Cyclosporin A	Multiple amides, Hydroxyl	Multiple IMHBs in folded structure	Yes
Atorvastatin	Carboxylic acid, lactone	Lactone form: internal H-bond	Lactone form penetrates
Sildenafil	Amide, pyrazole	Amide ↔ N acceptor	Yes

## Data Availability

Not applicable.
